# Mucus extravasation and retention phenomena: a 24-year study

**DOI:** 10.1186/1472-6831-10-15

**Published:** 2010-06-07

**Authors:** Alethea M Hayashida, Daniel CZ Zerbinatti, Ivan Balducci, Luiz Antonio G Cabral, Janete D Almeida

**Affiliations:** 1 Private Dentist, São Paulo, São Paulo, Brazil; 2 Department of Social Science and Pediatric Dentisty, São José dos Campos Dental School, São Paulo State University (UNESP), São José dos Campos, São Paulo, Brazil; 3 Department of Biosciences and Oral Diagnosis, São José dos Campos Dental School, São Paulo State University (UNESP), São José dos Campos, São Paulo, Brazil

## Abstract

**Background:**

Mucoceles are benign lesions related to the minor salivary glands and their respective ducts frequently affecting oral structures which are generally asymptomatic. Mucoceles are generally characterized by swollen nodular lesions preferentially located on the lower lip and differ from the so-called ranulas, which are lesions located on the floor of the mouth and related to the sublingual or submandibular glands.

**Methods:**

The objective of the present study was to analyze data such as age, gender, race and site of the lesion of 173 mucocele cases diagnosed at the Discipline of Stomatology, São José dos Campos Dental School, UNESP, over a period of 24 years (April 1980 to February 2003).

**Results:**

Of the 173 cases analyzed, 104 (60.12%) were females and 69 (39.88%) were males. Age ranged from 4 to 70 years (mean ± SD: 17 ± 9.53) and most patients were in the second decade of life (n = 86, 49.42%); white (n = 124, 71.68%). The lower lip was the site most frequently affected by the lesions (n = 135, 78.03%), whereas the lowest prevalence was observed for the soft palate, buccal mucosa, and lingual frenum.

**Conclusion:**

In this study, mucoceles predominated in white female subjects in the second decade of life, with the lower lip being the most frequently affected site.

## Background

Mucoceles are common minor salivary gland lesions clinically characterized by single or multiple, spherical, fluctuant nodules which are generally asymptomatic [1,2]. They are believed to result from mechanical trauma to the excretory duct of the salivary glands, causing duct transection or rupture, with consequent extravasation of mucin to the connective tissue stroma (mucus extravasation phenomenon, MEP). In addition, mucus might be retained in the duct and/or acinus as a result of duct obstruction (mucus retention phenomenon, MRP). MRP are less frequent and are seen particularly in the elderly [3-5].

Mucus extravasation triggers a secondary inflammatory reaction predominantly consisting of mononuclear cells in surrounding connective tissue, followed by a granulation tissue-type reaction that culminates in the formation of a fibrous capsule around the mucin deposit, conferring a cyst-like aspect to the lesion [6,7]. The diameter of mucoceles ranges from a few millimeters to centimeters [7,8]. Many patients report the periodic discharge of viscous fluid from the lesion.

According to several studies, the lower lip is the region most affected by mucoceles [2,6,9-15]. However, rare cases of mucoceles involving the upper lip, palate, retromolar region, buccal mucosa, lingual frenum, and dorsal tongue have been reported [5,8,11-16].

Ranula designates mucoceles located on the floor of the mouth [17]. The name is derived from the Latin word "rana" (meaning frog) because of its resemblance with the underbelly of a frog [1,2,5,6,8,9,11,13-15,18]. Ranulas are generally related to the duct systems of the sublingual salivary glands and, less frequently, to the submandibular gland and minor salivary gland ducts of the floor of the mouth [1]. A ranula manifests as a cup-shaped fluctuant bluish swelling on the floor of the mouth. Ranulas tend to be larger than mucoceles located in other regions of the mouth, reaching some centimeters in diameter. Depending on size and location, patient may present external swelling and relate discomfort, interference with speech, mastication, and swallowing [11].

The objective of the present study was to analyze cases of mucocele diagnosed between April 1980 and February 2003 at the Discipline of Semiology, São José dos Campos Dental School (FOSJC), UNESP, and to establish the prevalence of these lesions according to age, gender, race and site of ocurrence.

## Methods

In a survey of the clinical records of the Discipline of Stomatology, Department of Biosciences and Oral Diagnosis, São José dos Campos Dental School, São Paulo State University, comprising the period between April 1980 and February 2003, 173 cases with a diagnosis of MEP or MRP were selected. Data regarding age, gender, race and site of the lesion were obtained from these records. The study was approved by the Ethics Committee of FOSJC-UNESP (protocol 037/2003 PH/CEP).

## Results

**Figure 1:**
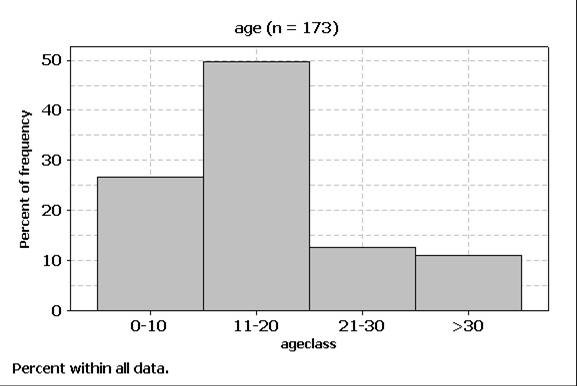
**Distribution according to age of cases of mucoceles diagnosed between April 1980 and February 2003 at the Discipline of Stomatology Department of Biosciences and Oral Diagnosis, São José dos Campos Dental School, São Paulo State University**.

Age ranged from 0 to 10 years in 46 (26.43%) patients, from 11 to 20 years in 86 (49.42%), from 21 to 30 in 22 (12.64%), and 19 (10.91%) subjects were older than 30 years, with a mean age of 17 years and a median age of 14 years (StDev = 11.341; minimum = 4; maximun = 70; Q1 = 10; Q3 = 21) (Figure 1).

Of the 173 cases studied, 104 (60.12%) were females and 69 (39.88%) were males. Regarding race, 124 subjects (71,68%) were white, 45 (26,01%) were black and four (2,31) were of Asian origin.

**Figure 2:**
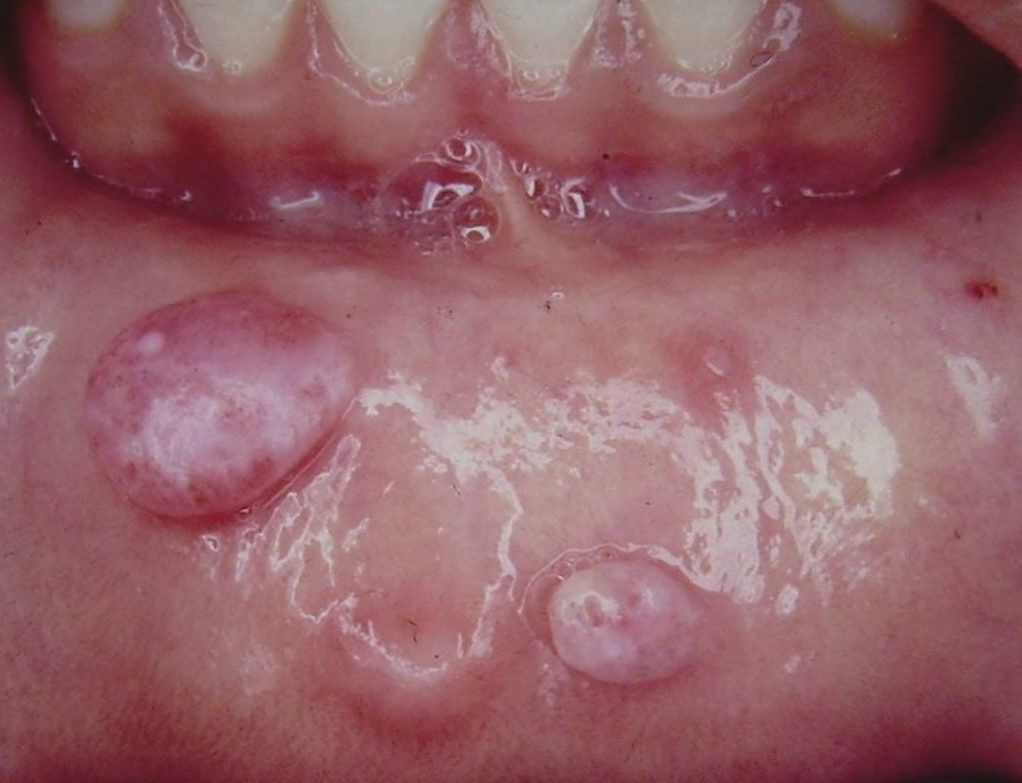
**Turgid nodular lesions located on the lower lip**.

**Figure 3:**
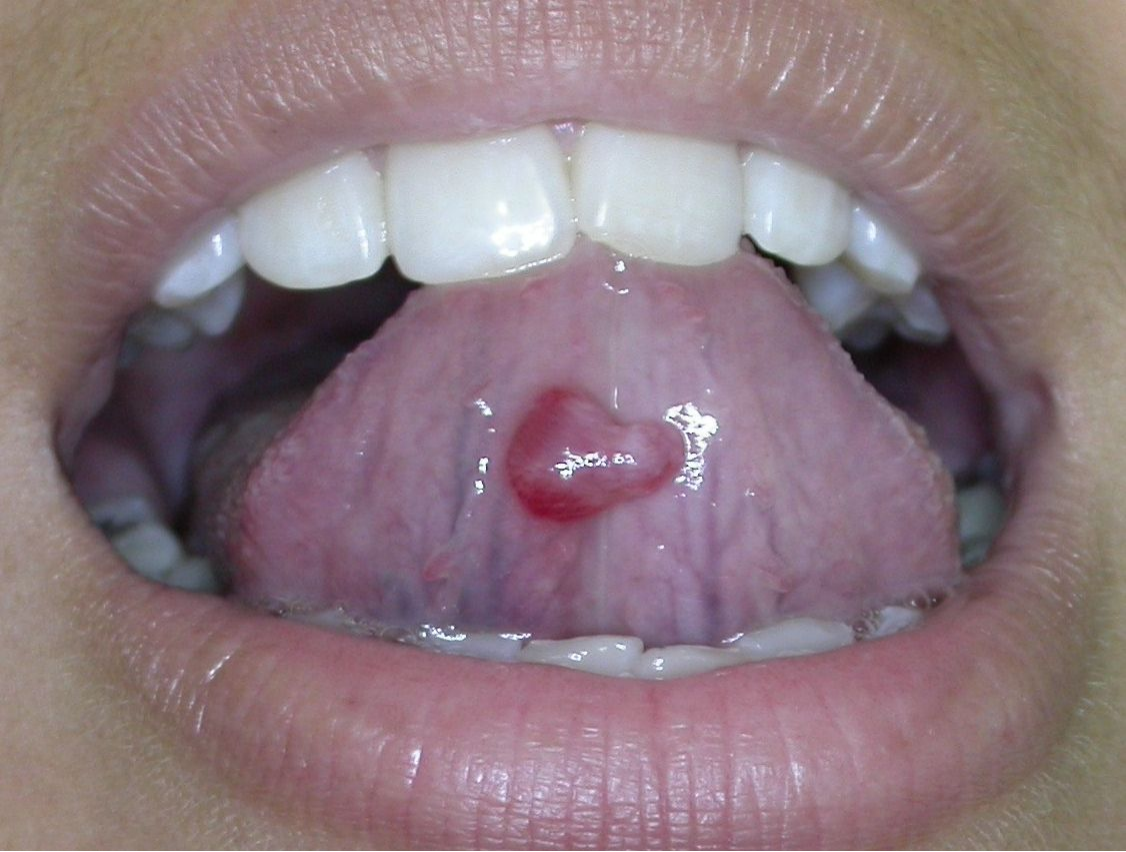
**Mucocele of the gland of Blandin-Nuhn (ventral tongue)**. Pediculated turgid nodular lesion, measuring 3.0 cm in diameter.

**Figure 4:**
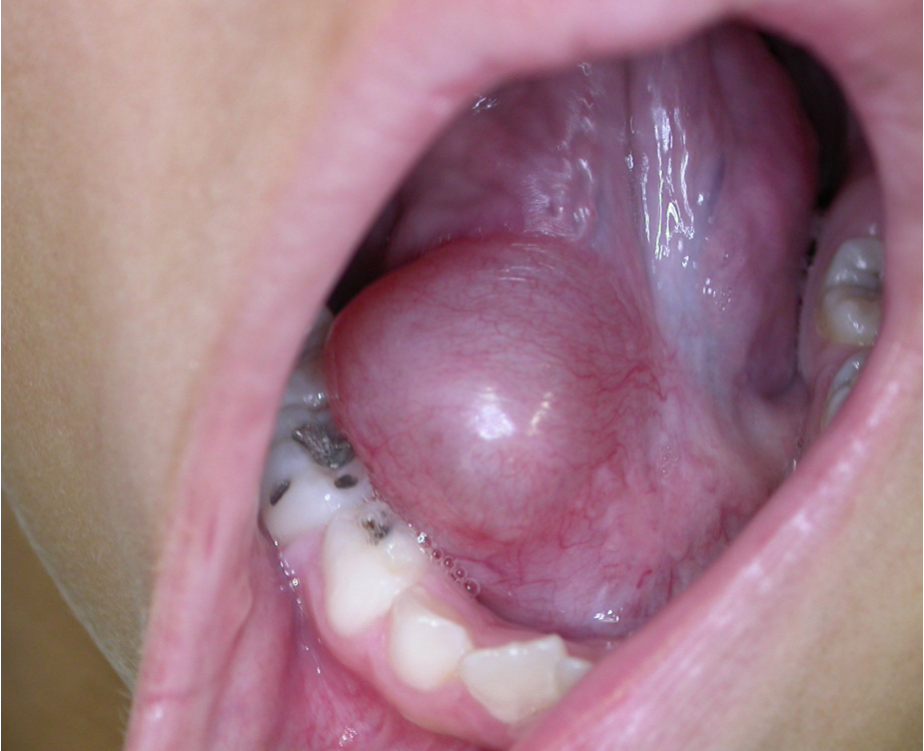
**Asymptomatic turgid nodular lesion located on the floor of the mouth, measuring 4.0 cm in diameter**.

**Figure 5:**
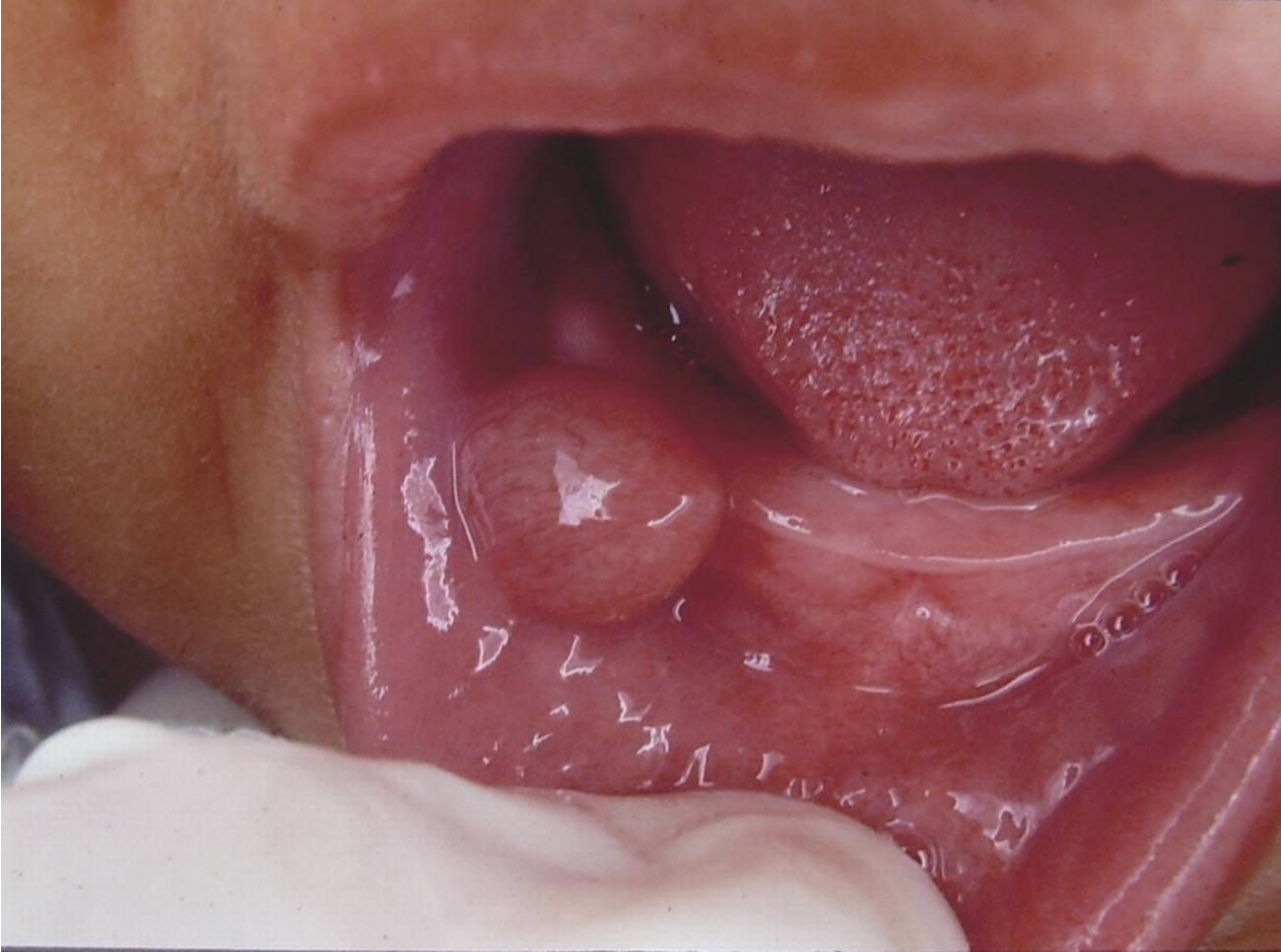
**Turgid nodular lesion located on the lower lip of an infant**.

Mucoceles were located on the lower lip in 78% of cases (Figure 2), on the ventral tongue in 9.83% (Figure 3), on the floor of the mouth in 9.25% (Figure 4), and on the soft palate, buccal mucosa (Figure 5) and lingual frenum in 0.58% each.

## Discussion

In general, salivary gland diseases can be subdivided into neoplastic and non-neoplastic diseases. The latter category includes different diseases that pose a diagnostic and therapeutic challenge to the clinician because of their closely similar clinical presentation despite different etiologies such as reactional inflammatory processes, metabolic and immune disorders, infections, and iatrogenic responses. Thus, clinical knowledge of oral lesions, as well as the determination of aspects related to the etiopathogenesis of these lesions, is necessary for the correct diagnosis and for the indication of appropriate treatment [16].

When located on the ventral tongue, the differential diagnosis with lymphangioma must be considered. de Camargo Moraes et al.[19] state that mucocele of the gland of Blandin-Nuhn (ventral tongue) should not be considered rare. In their series, this type of mucocele was the second most frequent.

Lesions located in the soft palate and retromolar region are rare, but in the latter case the differential diagnosis with mucoepidermoid carcinoma should be considered.

Among the non-neoplastic pathological processes affecting the minor salivary glands, mucoceles are the most common in children and young adults, a fact probably related to the higher frequency of injuries that result in extravasation of saliva to the adjacent connective tissue [11].

In agreement with similar studies reported in the literature, in the present investigation 75.85% of the cases were diagnosed during the first and second decades of life, 49.42% of them during the second decade of life. Two cases were diagnosed in newborns. Jones et al [9], analyzing 4406 children ranging in age from 0 to 16 years over a period of 30 years (1973-2002), observed 735 (16.68%) cases of mucoceles.

In the present study, most patients (60.12%) were females, in agreement with studies showing almost 70% prevalence of mucocele in women [17]. In contrast, Mathew et al. [20] describe a prevalence of mucocele in 0.16% of the population studied and the lesion was found only in males. Cataldo and Mosadomi [12], studying 594 cases between 1958 and 1969, observed no gender preference. As regards race, the lesion was more common in white subjects (124; 71,68%) in accordance to de Camargo Moraes et al. [19].

The most common location of mucoceles is the lower lip. This may be related to the trauma exerted upon the lip, as a result of teeth spatial distribution [3,11]. In the present study, mucoceles were observed on the lower lip in 78% of the cases, all presenting a history of trauma. Less frequently involved regions included the ventral tongue, floor of the mouth (ranula), hard and soft palate, buccal mucosa, and lingual frenum.

Mucoceles are more frequently treated by surgical excision of the lesion and careful dissection of the adjacent minor salivary glands affected [11,17]. However, recurrence can occur and a new surgical intervention taking the above mentioned care is necessary [11].

In the case of ranulas, treatment consists of surgical removal of the sublingual gland and/or marsupialization. Marsupialization may be performed before definitive excision of the gland in an attempt to permit the formation of an intraoral fistula through which saliva is excreted. This approach requires the removal of the roof of the lesion in order to permit reestablishment of the communication between the gland duct and oral cavity [11].

Yagüe-Garcia et al. [3] compared the results obtained after treatment with scalpel versus CO_2 _laser. Authors concluded that CO_2 _laser ablation is rapid and simple. They had postoperative complications and recurrence in the cases treated with conventional surgery. It is important to emphasize that the removed specimen must be microscopically evaluated to confirm the diagnosis, regardless of the technique used [3]. Prognosis is excellent.

## Conclusion

In this study, mucoceles predominated in white female subjects in the second decade of life, with the lower lip being the most frequently affected site.

## Competing interests

The authors declare that they have no competing interests.

## Authors' contributions

AMH and DCZZ analyzed and interpreted patient's data from the files. IB performed the statistical analysis. LAGC participated in the design of the research. JDA conceived, coordinated and helped to draft the manuscript. All authors read and approved the final manuscript. All authors read and approved the final manuscript.

## Pre-publication history

The pre-publication history for this paper can be accessed here:

http://www.biomedcentral.com/1472-6831/10/15/prepub
